# Supplementary material for: Numerical approaches for the rapid analysis of prophylactic efficacy against HIV with arbitrary drug-dosing schemes
Source: PLoS Comput Biol. 2021 Dec 23;17(12):e1009295. doi: 10.1371/journal.pcbi.1009295 (PMC8741042; doi:10.1371/journal.pcbi.1009295)
Supplement: S1 Text — (PDF) [file pcbi.1009295.s001.pdf]

S1 Text

Derivation of the distribution of state transition events

For the transition $T_1 \rightarrow T_2$, the probability distribution $f_{a_5}(x)$ cannot be computed easily since the values of a_5 are time-dependent. Because a_5 represents the expectation of how many times the transition $T_1 \rightarrow T_2$ occurs in an infinitesimal duration, for a very short time span Δt, such that $a_5 \Delta t << 1$; $a_5 \Delta t$ can be treated as the probability that $T_1 \rightarrow T_2$ happens in Δt. Therefore, the distribution $f_{a_5}(x)$ can be represented as follows:

$$\begin{aligned} f_{a_5}(x) \cdot \Delta t &= \lim_{\Delta t \to 0} (1 - a_5(t_0)\Delta t) \cdot (1 - a_5(t_1)\Delta t) \cdot \ldots \cdot (1 - a_5(t_{n-1})\Delta t) \cdot a_5(x)\Delta t \\ &= \lim_{\Delta t \to 0} \prod_{i=0}^{n-1} (1 - a_5(t_i)\Delta t) \cdot a_5(x)\Delta t \end{aligned} \tag{S1.1}$$

where $t_0 = 0, t_n = x$ and $\Delta t \cdot n = x$. Applying a logarithmic transformation to the equation above gives:

$$\begin{aligned} \log f_{a_5}(x) &= \lim_{\Delta t \to 0} \log \left(\prod_{i=0}^{n} (1 - a_5(t_i)\Delta t) \cdot a_5(x) \right) \\ &= \lim_{\Delta t \to 0} \sum_{i=0}^{n} \log (1 - a_5(t_i)\Delta t) + \log a_5(x) \end{aligned}$$

For a very small time step $\Delta t \rightarrow 0$, the equation above can be represented as:

$$\log f_{a_5}(x) = \int_0^x \log (1 - a_5(t)dt) + \log a_5(x)$$

The first term can be approximated by applying a first-order Taylor expansion around $a_5(t)dt = 0$:

$$\log (1 - a_5(t)dt) \approx \log (1 - 0) - \frac{1}{(1-0)} (a_5(t)dt - 0) = -a_5(t)dt$$

and therefore:

$$f_{a_5}(x) = a_5(x) e^{-\int_0^x a_5(t)dt} \tag{S1.2}$$

Now, the probability distribution for the transition $f_{T_1 \rightarrow T_2}(x)$ can be written as:

$$f_{T_1 \rightarrow T_2}(x) = (1 - F_{a_2}(x)) \cdot f_{a_5}(x) = a_5(x) e^{-(a_2 x + \int_0^x a_5(t)dt)} \tag{S1.3}$$

Probability Generating System (PGS)

In the discrete form, the extinction probability $P_E\left(Y_t = \hat{V}\right)$ is derived as follows:

$$\begin{aligned} P_E\left(Y_t = \hat{V}\right) =& P\left(Y_{t+\Delta t} = \mathbf{0} \mid Y_t = \hat{V}\right) + \\ & P\left(Y_{t+\Delta t} = \hat{T}_1 \mid Y_t = \hat{V}\right) \cdot P_E\left(Y_{t+\Delta t} = \hat{T}_1\right) + \\ & P\left(Y_{t+\Delta t} = \hat{V} \mid Y_t = \hat{V}\right) \cdot P_E\left(Y_{t+\Delta t} = \hat{V}\right) \end{aligned} \tag{S1.4}$$

Each term in this equation can be derived based on the distributions of state transition events:

$$\begin{aligned} P\left(Y_{t+\Delta t} = \mathbf{0} \mid Y_t = \hat{V}\right) &= \int_t^{t+\Delta t} (1 - F_{a_4}(x-t)) \cdot f_{a_1}(x-t)dx \\ &= \int_t^{t+\Delta t} e^{-a_4(x-t)} \cdot a_1 e^{-a_1(x-t)}dx \\ &= \int_t^{t+\Delta t} a_1 \cdot e^{-(a_1+a_4)(x-t)}dx \\ &= -\frac{a_1}{a_1+a_4} e^{-(a_1+a_4)(x-t)} \mid_t^{t+\Delta t} \\ &= -\frac{a_1}{a_1+a_4}\left(e^{-(a_1+a_4)\Delta t} - 1\right) \end{aligned}$$

$$\begin{aligned} P\left(Y_{t+\Delta t} = \hat{T}_1 \mid Y_t = \hat{V}\right) &= \int_t^{t+\Delta t} f_{V \to \mathrm{T}_1}(x-t)dx \\ &= \int_t^{t+\Delta t} a_4 e^{-(a_1+a_4)(x-t)}dx \\ &= -\frac{a_4}{a_1+a_4} e^{-(a_1+a_4)(x-t)} \mid_t^{t+\Delta t} \\ &= -\frac{a_4}{a_1+a_4}\left(e^{-(a_1+a_4)\Delta t} - 1\right) \end{aligned}$$

$$\begin{aligned} P\left(Y_{t+\Delta t} = \hat{V} \mid Y_t = \hat{V}\right) &= 1 - P\left(Y_{t+\Delta t} = \varnothing \mid Y_t = \hat{V}\right) \\ &\quad - P\left(Y_{t+\Delta t} = \hat{T}_1 \mid Y_t = \hat{V}\right) \\ &= 1 + \frac{a_1}{a_1+a_4}\left(e^{-(a_1+a_4)\Delta t} - 1\right) \\ &\quad + \frac{a_4}{a_1+a_4}\left(e^{-(a_1+a_4)\Delta t} - 1\right) \\ &= e^{-(a_1+a_4)\Delta t} \end{aligned}$$

Plugging these three terms into eq (S1.5):

$$\begin{aligned} P_E\left(Y_t=\hat{V}\right)= & \frac{a_1}{a_1+a_4}\left(1-e^{-(a_1+a_4)\Delta t}\right) \\ & + \frac{a_4}{a_1+a_4}\left(1-e^{-(a_1+a_4)\Delta t}\right)\cdot P_E\left(Y_{t+\Delta t}=\hat{T}_1\right) \\ & + e^{-(a_1+a_4)\Delta t}\cdot P_E\left(Y_{t+\Delta t}=\hat{V}\right) \end{aligned} \tag{S1.5}$$

$P\left(\varnothing \mid Y_t=\hat{T}_1\right)$ and $P\left(\varnothing \mid Y_t=\hat{T}_2\right)$ can be derived analogously.

Based on the eqs.(24)–(26), if Δt is small enough, a first-order Taylor expansion around $\Delta t=0$ can be applied: $e^{-(a_1+a_4)\Delta t}\approx 1-(a_1+a_4)\Delta t$. Then, eq.(24) can be transformed into:

$$\begin{aligned} P_E\left(Y_t=\hat{V}\right) &= a_1\Delta t + a_4\Delta t\cdot P_E\left(Y_{t+\Delta t}=\hat{T}_1\right) \\ &\quad + (1-(a_1+a_4)\Delta t)\cdot P_E\left(Y_{t+\Delta t}=\hat{V}\right) \\ &= P_E\left(Y_{t+\Delta t}=\hat{V}\right) - (a_1+a_4)\Delta t\cdot P_E\left(Y_{t+\Delta t}=\hat{V}\right) \\ &\quad + a_1\Delta t + a_4\Delta t\cdot P_E\left(Y_{t+\Delta t}=\hat{T}_1\right). \end{aligned} \tag{S1.6}$$

The derivative of $P_E\left(Y_t=\hat{V}\right)$ can then be expressed as:

$$\begin{aligned} \frac{dP_E\left(Y_t=\hat{V}\right)}{dt} &= \lim_{\Delta t\to 0}\frac{P_E\left(Y_{t+\Delta t}=\hat{V}\right)-P_E\left(Y_t=\hat{V}\right)}{\Delta t} \\ &= \lim_{\Delta t\to 0}(a_1+a_4)\cdot P_E\left(Y_{t+\Delta t}=\hat{V}\right) \\ &\qquad - \lim_{\Delta t\to 0}\left(a_1+a_4\cdot P_E\left(Y_{t+\Delta t}=\hat{T}_1\right)\right) \\ &= a_1\cdot\left(P_E\left(Y_t=\hat{V}\right)-1\right) \\ &\qquad + a_4\cdot\left(P_E\left(Y_t=\hat{V}\right)-P_E\left(Y_t=\hat{T}_1\right)\right). \end{aligned} \tag{S1.7}$$

Equations for $\frac{dP_E\left(Y_t=\hat{T}_1\right)}{dt}$ and $\frac{dP_E\left(Y_t=\hat{T}_2\right)}{dt}$ can be derived accordingly.
