# Supplementary material for: Numerical approaches for the rapid analysis of prophylactic efficacy against HIV with arbitrary drug-dosing schemes
Source: PLoS Comput Biol. 2021 Dec 23;17(12):e1009295. doi: 10.1371/journal.pcbi.1009295 (PMC8741042; doi:10.1371/journal.pcbi.1009295)
Supplement: S2 Text — (PDF) [file pcbi.1009295.s002.pdf]

S2 Text

Implementation and pseudo-code of Probability Generating System (PGS)

The PGS computes the extinction probabilities *backwards in time*, but uses an ODE solver to do so. Modern ODE-solvers automatically adapt the time step to achieve a default-, or user-defined accuracy. Hence, the time-step does not need to be defined *a priori*, but depends on the tolerance of the ODE-solver. The pseudo-code of the PGS is shown in Algorithm 1.

- Precomputation:
  - Define an extra time τ that has to be added to the time interval of interest $[T_s, T_e]$. In our predictions we use $\tau = 100\text{hours} \approx 7 \cdot t_{1/2}$, where $t_{1/2} \approx 14.5\text{hours}$ denotes the half life of DTG. This choice of τ guarantees that the concentrations of the drug at time $T_e + \tau$ are $< 1\%$ of the drug's trough levels.
  - Pre-compute the pharmacokinetic profile D_t for time interval $[T_s, T_e + \tau]$ by solving eq. (11)-(13) (main article). The pharmacokinetic solution can be packed into a function so that the drug concentration at any time point within the interval $[T_s, T_e + \tau]$ can be called.
  - The reaction propensities for the single educt molecules are pre-computed. Propensity a_5 should also be time-continuous like D_t, and could be called "on the fly" using the function for D_t.
  - Pre-calculate the extinction probabilities $P_E(Y_t = \hat{V}, \varnothing)$, $P_E(Y_t = \hat{T}_1, \varnothing)$ and $P_E(Y_t = \hat{T}_2, \varnothing)$, for the three unit vectors in eq. (4) (main manuscript), in the absence of drugs, e.g. given in [1].
- Initialization:
  - The initial values of the ODE (eq. (23), main manuscript) are set to $[P_E(Y_t = \hat{V}, \varnothing), P_E(Y_t = \hat{T}_1, \varnothing), P_E(Y_t = \hat{T}_2, \varnothing)]$ and the time point to begin the integration to $T_e + \tau$.

The set of ODEs eq. (23) (main manuscript is solved backwards: in this work we used the ODE solver `solve_ivp` in `SciPy`. The function to compute the pharmacokinetic profile D_t, was called within the function that was passed to the ODE-solver, so that the value of a_5 can be updated during the integration process.

Algorithm 1: Probability Generating System

Input: start time point T_s, end time point $T_e + \tau$

Result: extinction probabilities profile $P_E\left(Y_t = \hat{V}, \mathcal{S}\right)$, $P_E\left(Y_t = \hat{T}_1, \mathcal{S}\right)$ and $P_E\left(Y_t = \hat{T}_2, \mathcal{S}\right)$

Precomputation:

compute target-site pharmacokinetic profile D_t for $t \in (T_s, T_e + \tau)$;

compute values of a_5 based on D_t: $a_5(t)$ for $Y_t = \hat{T}_1$;

compute the reaction probabilities a_j, $j \in \{1, 2, 3, 4, 6\}$ for single educt molecules respectively in the absence of drugs;

compute the extinction probabilities in the absence of drugs: $P_E(Y_t = \hat{V}, \varnothing)$, $P_E(Y_t = \hat{T}_1, \varnothing)$, $P_E(Y_t = \hat{T}_2, \varnothing)$

Solve the ODE set with initial value

Initialization: Time span $[T_s, T_e + \tau]$, initial values: $[P_E(Y_t = \hat{V}, \varnothing)$, $P_E(Y_t = \hat{T}_1, \varnothing)$, $P_E(Y_t = \hat{T}_2, \varnothing)]$ at time point $T_e + \tau$

Solve the ODEs *backwards*:

$$\begin{aligned}
\frac{dP_E(Y = \hat{V})}{dt} &= a_1 \cdot \Big(P_E(Y = \hat{V}) - 1\Big) + a_4 \cdot \Big(P_E(Y = \hat{V}) - P_E(Y = \hat{T}_1)\Big) \\
\frac{dP_E(T = \hat{T}_1)}{dt} &= a_2 \cdot \Big(P_E(Y = \hat{T}_1) - 1\Big) + a_5(t) \cdot \Big(P_E(Y = \hat{T}_1) - P_E(Y = \hat{T}_2)\Big) \\
\frac{dP_E(Y = \hat{T}_2)}{dt} &= a_3 \cdot \Big(P_E(Y = \hat{T}_2) - 1\Big) \\
&\quad + a_6 \cdot \Big(P_E(Y = \hat{T}_2) - P_E(Y = \hat{T}_2) \cdot P_E(Y = \hat{V})\Big)
\end{aligned}$$

References

[1] Duwal S, Dickinson L, Khoo S, von Kleist M. Mechanistic framework predicts drug-class specific utility of antiretrovirals for HIV prophylaxis. PLoS Comput Biol. 2019;15(1):e1006740. doi:10.1371/journal.pcbi.1006740.
