# Supplementary material for: Numerical approaches for the rapid analysis of prophylactic efficacy against HIV with arbitrary drug-dosing schemes
Source: PLoS Comput Biol. 2021 Dec 23;17(12):e1009295. doi: 10.1371/journal.pcbi.1009295 (PMC8741042; doi:10.1371/journal.pcbi.1009295)
Supplement: S3 Text — (PDF) [file pcbi.1009295.s003.pdf]

S3 Text

PGS for extended viral dynamics model with latently infected cells

It has been shown that viral reservoirs may be a major barrier to viral extinction. Moreover, it has been shown that these reservoirs establish early after viral infection [1, 2, 3]. Once a reservoir is established, infection may be considered irreversible [4]. Based on a previous study [4], we also derived the PGS for an extended viral dynamics model that considers HIV reservoirs, as shown in Fig S3.1. This dynamics of the considered model can be defined by the following

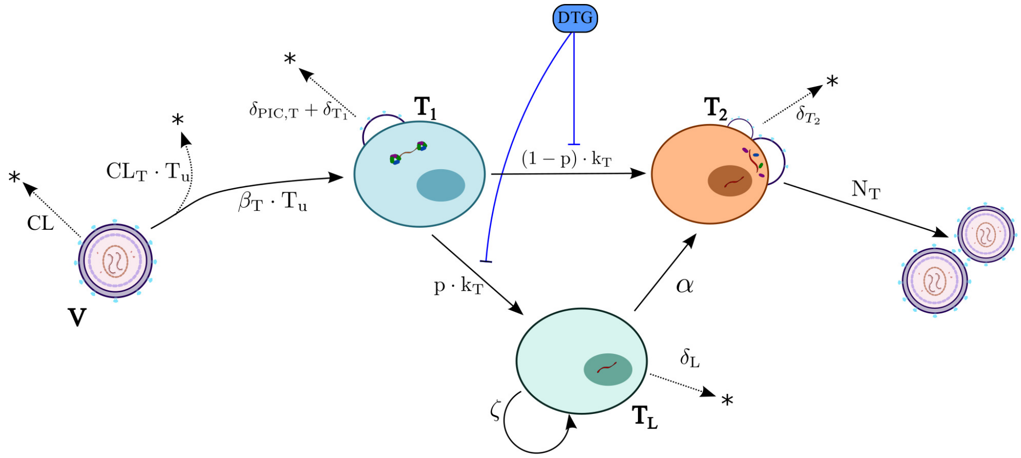

Figure S3.1: **Extended viral dynamics with latently infected cells.** Free viruses are cleared by the immune system with a constant rate CL. Further, viruses can also be cleared during unsuccessful T-cell infection $\mathrm{CL_T}$. The terms β_{T} represents the lumped rate of infection of T-cells. Early infected cells $\mathrm{T_1}$ can also be cleared with rate $\delta_{\mathrm{T_1}}$, or through the intracellular destruction of the pre-integration complex, with rate $\delta_{\mathrm{PIC,T}}$. The viral DNA can integrate to the host genome with rate $(1-p)\cdot \mathrm{k_T}$, transforming the cells into productively infected $\mathrm{T_2}$ cells. With rate $p \cdot \mathrm{k_T}$ latently infected cells $\mathrm{T_L}$ may emerge during- or shortly after the integration step, most likely due to chromatin rearrangement. These latently infected cells $\mathrm{T_L}$ do not produce new viruses, but can be reactivated (e.g. by subsequent chromatin rearrangement) into a productively infected $\mathrm{T_2}$ cells with rate α. The latently infected cells may also proliferate with rate ζ. The clearance rate of $\mathrm{T_2}$ and $\mathrm{T_L}$ cells are $\delta_{\mathrm{T_2}}$ and δ_{L}, respectively. $\mathrm{T_2}$ cells release new viruses with rate $\mathrm{N_T}$.

10 reactions:

R_1 : Clearance of free virus, V $\to *$ $\quad a_1 = (CL + CL_{\mathrm{T}} \cdot T_u) \cdot V$

R_2 : Clearance of T_1-cell, $T_1 \to *$ $\quad a_2 = (\delta_{\mathrm{PIC,T}} + \delta_{\mathrm{T_1}}) \cdot T_1$

R_3 : Clearance of T_2-cell, $T_2 \to *$ $\quad a_3 = \delta_{\mathrm{T_2}} \cdot T_2$

R_4 : Infection of a suscept. cell, $V \to \mathrm{T_1}$ $\quad a_4 = \beta_{\mathrm{T}} \cdot T_u \cdot V$

R_5 : Integration of viral DNA in T-cell, $\mathrm{T_1} \to \mathrm{T_2}$ $\quad a_5(\varnothing) = (1-p) \cdot k_{\mathrm{T}} \cdot T_1$

R_6 : T cells produce new virus, $\mathrm{T_2} \to V + \mathrm{T_2}$ $\quad a_6 = N_{\mathrm{T}} \cdot T_2$

R_7 : Generation of latently infected cell, $\mathrm{T_1} \to \mathrm{T_L}$ $\quad a_7(\varnothing) = p \cdot k_{\mathrm{T}} \cdot T_1$

R_8 : Reactivation of latently infected cell, $\mathrm{T_L} \to \mathrm{T_2}$ $\quad a_8 = \alpha \cdot T_L$

R_9 : Proliferation of latently infected cell, $\mathrm{T_L} \to \mathrm{T_L} + \mathrm{T_L}$ $\quad a_9 = \zeta \cdot T_L$

R_{10} : Clearance of latently infected cell, $\mathrm{T_L} \to *$ $\quad a_{10} = \delta_L \cdot \mathrm{T_L}$

with parameters given in Table S3.1.

Table S3.1: **Parameters for viral dynamics model.**

**Parameter**	**Value**	**Reference**	**Parameter**	**Value**	**Reference**
CL	2.3	[5, 6]	CL_{T}	$CL_{\mathrm{T}} = \left(\frac{1}{\rho_{rev}} - 1\right) \cdot \beta_{\mathrm{T}}$	[7]
T_u	$T_u = \lambda_{\mathrm{T}}/\delta_{\mathrm{T}}$		ρ_{rev}	0.5	[8, 9]
β_{T}	$8 \cdot 10^{-12}$	[10]	λ_{T}	$2 \cdot 10^9$	[11]
δ_{T}	0.02	[12]	$\delta_{\mathrm{PIC,T}}$	0.35	[9, 13]
$\delta_{\mathrm{T_1}}$	0.02	[12]	$\delta_{\mathrm{T_2}}$	1	[14]
k_{T}	0.35	[9]	N_{T}	670	[7, 12]
p	$8 \cdot 10^{-6}$	[15]	α	10^{-3}	[15]
ζ	0.047	[16]	δ_L	10^{-4}	[15, 17]

All parameters are in units [1/day], except for λ [cells/day] and β [1/(day·virus)]

The equations for the probability generating system (PGS) can be adapted to the extended viral dynamics model in Fig. S3.1 to derive:

$$\begin{aligned}
\frac{dP_E(Y=\hat{V})}{dt} &= (a_1 + a_4) \cdot P_E(Y=\hat{V}) - a_4 \cdot P_E(Y=\hat{T}_1) - a_1 \\
\frac{dP_E(Y=\hat{T}_1)}{dt} &= (a_2 + a_5(t) + a_7(t)) \cdot P_E(Y=\hat{T}_1) - a_5(t) \cdot P_E(Y=\hat{T}_2) \\
&\quad - a_7(t) \cdot P_E(Y_t=\hat{T}_L) - a_2 \\
\frac{dP_E(Y=\hat{T}_2)}{dt} &= (a_3 + a_6) \cdot P_E(Y=\hat{T}_2) - a_6 \cdot P_E(Y=\hat{T}_2) \cdot P_E(Y=\hat{V}) - a_3 \\
\frac{dP_E(Y_t=\hat{T}_L)}{dt} &= (a_8 + a_9 + a_{10}) \cdot P_E(Y_t=\hat{T}_L) - a_8 \cdot P_E(Y=\hat{T}_2) \\
&\quad - a_9 \cdot P_E(Y_t=\hat{T}_L)^2 - a_{10}
\end{aligned} \tag{S1.1}$$

Effect of latent reservoirs on the extinction probability. Fig. S3.2 shows the extinction probability profiles for a 3 days 50mg oral DTG regimen, using same pharmacokinetic parameters as in Fig. 3 (main manuscript). Comparing the extinction probability courses $P_E(Y_t = \hat{V}, \mathcal{S})$, $P_E(Y_t = \hat{T}_1, \mathcal{S})$ and $P_E(Y_t = \hat{T}_2, \mathcal{S})$ using the simple viral dynamics model (Fig. 1, main manuscript) vs. the extended viral dynamics model that includes the latently infected cells (Fig. S3.2 herein), we get negligible differences. Quantitatively, differences in $P_E(Y_t = \hat{V}, \mathcal{S})$ between the two models are of the order $10^{-5} - 10^{-4}$ ($0.001\% - 0.01\%$). **Our results therefore demonstrate that the impact of the latent reservoirs on prophylactic efficacy is negligible**, and hence prophylactic efficacy can be well estimated using the model presented in the main manuscript.

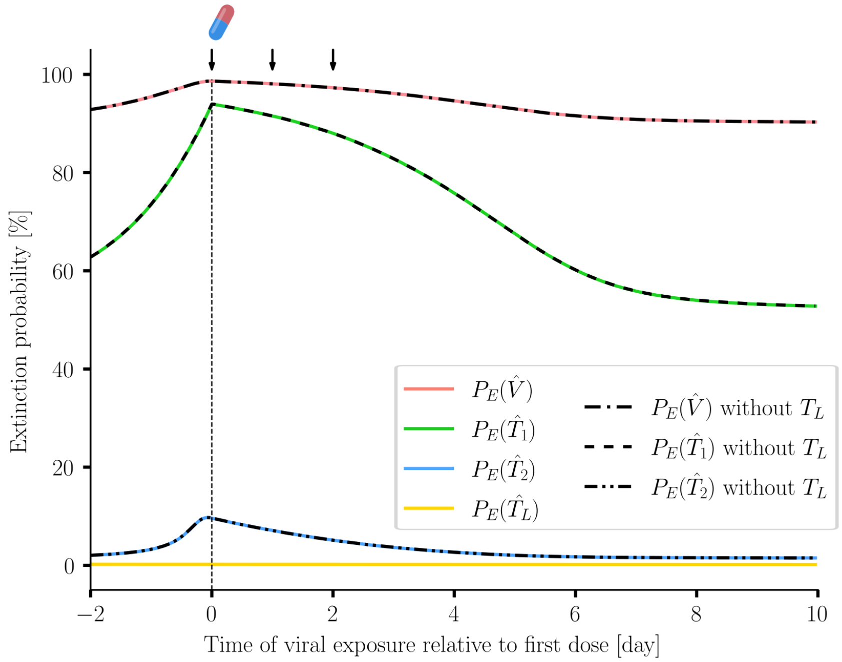

Figure S3.2: **Extinction probability profiles based on a viral dynamics model containing latently infected cells**. The solid colored lines show the extinction probabilities for the extended viral dynamics model (Fig. S3.1) for 3-days once daily short-course oral 50mg DTG regimen, whereas the dashed black lines show the corresponding profiles using the simpler model presented in the main manuscript, which does not contain latently infected cells.

References

[1] Chun TW, Moir S, Fauci AS. HIV reservoirs as obstacles and opportunities for an HIV cure. Nature immunology. 2015;16(6):584–589.

[2] Chun TW, Engel D, Berrey MM, Shea T, Corey L, Fauci AS. Early establishment of a pool of latently infected, resting CD4+ T cells during primary HIV-1 infection. Proceedings of the National Academy of Sciences. 1998;95(15):8869–8873.

[3] Whitney JB, Hill AL, Sanisetty S, Penaloza-MacMaster P, Liu J, Shetty M, et al. Rapid seeding of the viral reservoir prior to SIV viraemia in rhesus monkeys. Nature. 2014;512(7512):74–77.

[4] Duwal S, Dickinson L, Khoo S, von Kleist M. Hybrid stochastic framework predicts efficacy of prophylaxis against HIV: An example with different dolutegravir prophylaxis schemes. PLOS Computational Biology. 2018;14(6):e1006155.

[5] Tuckwell HC, Shipman PD, Perelson AS. The probability of HIV infection in a new host and its reduction with microbicides. Mathematical Biosciences. 2008;214(1-2):81–86.

[6] Tan WY, Wu H. Stochastic modeling of the dynamics of CD4+ T-cell infection by HIV and some Monte Carlo studies. Mathematical Biosciences. 1998;147(2):173–205.

[7] Von Kleist M, Menz S, Huisinga W. Drug-class specific impact of antivirals on the reproductive capacity of HIV. PLOS Computational Biology. 2010;6(3):e1000720.

[8] Pierson TC, Zhou Y, Kieffer TL, Ruff CT, Buck C, Siliciano RF. Molecular characterization of preintegration latency in human immunodeficiency virus type 1 infection. Journal of virology. 2002;76(17):8518–8531.

[9] Zhou Y, Zhang H, Siliciano JD, Siliciano RF. Kinetics of human immunodeficiency virus type 1 decay following entry into resting CD4+ T cells. Journal of Virology. 2005;79(4):2199–2210.

[10] Sedaghat AR, Dinoso JB, Shen L, Wilke CO, Siliciano RF. Decay dynamics of HIV-1 depend on the inhibited stages of the viral life cycle. Proceedings of the National Academy of Sciences. 2008;105(12):4832–4837.

[11] Wei X, Ghosh SK, Taylor ME, Johnson VA, Emini EA, Deutsch P, et al. Viral dynamics in human immunodeficiency virus type 1 infection. Nature. 1995;373(6510):117–122.

[12] Sedaghat AR, Siliciano RF, Wilke CO. Constraints on the dominant mechanism for HIV viral dynamics in patients on raltegravir. Antiviral Therapy. 2009;14(2):263.

[13] Koelsch KK, Liu L, Haubrich R, May S, Havlir D, Günthard HF, et al. Dynamics of total, linear nonintegrated, and integrated HIV-1 DNA in vivo and in vitro. The Journal of Infectious Diseases. 2008;197(3):411–419.

[14] Markowitz M, Louie M, Hurley A, Sun E, Di Mascio M, Perelson AS, et al. A novel antiviral intervention results in more accurate assessment of human immunodeficiency virus type 1 replication dynamics and T-cell decay in vivo. Journal of Virology. 2003;77(8):5037–5038.

[15] Callaway DS, Perelson AS. HIV-1 infection and low steady state viral loads. Bulletin of mathematical biology. 2002;64(1):29–64.

[16] Reeves DB, Duke ER, Wagner TA, Palmer SE, Spivak AM, Schiffer JT. A majority of HIV persistence during antiretroviral therapy is due to infected cell proliferation. Nature communications. 2018;9(1):1–16.

[17] Curlin ME, Iyer S, Mittler JE. Optimal timing and duration of induction therapy for HIV-1 infection. PLoS computational biology. 2007;3(7):e133.
