# Supplementary material for: Numerical approaches for the rapid analysis of prophylactic efficacy against HIV with arbitrary drug-dosing schemes
Source: PLoS Comput Biol. 2021 Dec 23;17(12):e1009295. doi: 10.1371/journal.pcbi.1009295 (PMC8741042; doi:10.1371/journal.pcbi.1009295)
Supplement: Attachment — Submitted filename: ReplyLetter.pdf [file pcbi.1009295.s004.pdf]

We thank the editor and reviewers for their time and constructive comments, which improved the manuscript. Below is a point-by-point response to all reviewers' comments. Based on the reviewer's feedback we conducted the following major changes and reorganised the manuscript accordingly:

- We tested our numerical method using the model extensions (dynamics of latently infected cells) suggested (Rev # 1, comment 1) and show the results in the novel Supplementary Text S3.
- We shortened the manuscript considerably, focusing on the third method (PGS, Rev # 2, comment 1). This led to some re-arrangements of the manuscript. Besides deleting the corresponding passages in the *Methods* and *Results* section and adapting the text in the remainder of the manuscript, the following changes were conducted to the supplement and figures/tables:

Supplementary Texts		Figures & Tables	
**original**	**revised**	**original**	**revised**
S2	deleted	-	Fig. 2
S3	deleted	Fig.2–6	Fig. 3–7
S4	S2	Table 2	deleted
S5	S3 (revised)	Table 3	Table 2
S6	deleted		

Reviewer #1: A few comments for the authors to consider. Overall, the model is overly simple for such complicated disease like HIV. The main issue is no latent reservoir is modeled in the primary model. Latent reservoir is critical for HIV eradication, it's also critical for PrEP. Once the HIV latent reservoir is seeded, there is no way HIV infection can go extinction with current standard-of-care, including DTG. An earlier study showed the latent reservoir seeding can happen as soon as within hours to a few days after initial infection (Whitney et al. Nature 2014 (512):74-77). And many aspects of the HIV infection biology are not yet understood, but it is likely more complicated than the model in this paper.
The authors claimed in the discussions that adding long-lived or latent reservoir to the model seems not impacting the result much. However, the latent reservoir model that the authors described in the Fig S5.1 does not have latency self-renewal and activation mechanism. That would allow HIV goes to extinct in the model, which in reality will never happen. Latently infected cells have great capacity to maintain itself from extinction, and it can become productively infected cell and reentry to the viral replication cycle stochastically, i.e. at least $T_L- > 2xT_L$ and $T_L- > T_2$ should be modeled in the system. Without these mechanisms, it is not surprising that adding latent reservoir is not impacting the results.
Focus of the paper: We appreciate the reviewers comment, but also want to point out that the **focus of this paper** was to introduce a new numerical

method that allows computing PrEP efficacy much faster, without the need of computationally expensive and excessive sampling. The focus of the paper is not on the underlying viral model (the utilized model had been introduced before (PMID: 20361047) and is thus merely used for validation purposes).

Role of the latent reservoir for PrEP. We agree with the reviewer that the latent reservoir is critical for HIV eradication (once infection has established), but we disagree that it is critical for pre-exposure prophylaxis (outlined below). We included a discussion on this topic (lines 523-532).

- We ran 1 million stochastic simulations using the settings and method from PMID:29902179, without drug and with one initial infectious virus. In that model, parameters (probability of the reservoir forming per cell infection) have been adjusted in previous work based on available clinical data, matching clinical dynamics of HIV (PMID:21455303). In line with previous work (PMID:29902179) stochastic simulations were counted as 'infection events' when trajectories left an extinction simplex (extinction unlikely due to viral expansion), or if a single long-lived or latently infected cell emerged (thus considering events even before latency self-renewal). In total, 98231 simulations were counted as 'infections' and of those, only 37 (0.037% of the infection simulations) were due to a long-lived or latent reservoir. Therefore, ignoring the reservoir when predicting the prophylactic efficacy appears statistically acceptable.

- To demonstrate that our method can be applied to arbitrary models, we performed the suggested model extensions, derived the PGS and estimate PrEP efficacy. The results of this model extensions are shown in the revised Supplementary Text S3. Here, we can see that the predictions change marginally, if infection starts with one virus V, one early infected T_1 or late infected T_2 target cell, even if there is self-renewal and activation of latently infected cells. As pointed out by the reviewer and also substantiated by us in the original submission, if a single latently infected cell arises, infection is certain (= elimination is impossible). The latter can be seen in Fig. S3.2 in Suppl. Text S3 ('starting from a single latently infected cell', yellow line). However, as can be seen, the contribution of these latently infected cell dynamics to $P_E(\hat{V})$ are marginal (i.e., the contribution of latently infected cell dynamics to the probability of extinction after exposure to free virus is negligible).

- Coming back to the literature suggested by the reviewer: We want to point out a few facts with regards to the interpretation of the suggested article: In the analyzed SIV-Macaque system, (i) the authors show that *a cellular reservoir* (not necessarily latently-infected [= quiecent] cells) is established *within the first 3 days* after infection. To analyse this, (ii) the authors use an extremely large inoculum (500 $TCID_{50}$) to ensure that the monkeys get infected. (iii) Subsequently, they start (post-exposure) treatment at day 3 after infection. Firstly, the study provides no direct proof that the cellular reservoir is indeed the *latent reservoir* (we could also

not find such a statement in the article). Secondly, the utilized inoculum is quite different to the typical human exposure (with sexual per-exposure infection risks of $0.3\% - 3\%$). The inoculum has consequences with regards to the ability to eliminate the virus *post*-exposure. Thirdly, considering the above statements, the article is actually fully in line with our model simulations [without a latent reservoir], e.g. as shown in Fig. 5 (left side, large inoculum sizes): With large inoculums, it is virtually impossible to prevent infection using post-exposure prophylaxis.

In summary, we strongly agree with the reviewer that the latent reservoir is important, particularly in making infection irreversible once it has *established*. Therefore, it is particularly important in preventing viral elimination during treatment of an established HIV infection. When looking at PrEP efficacy, one actually considers events prior to that: What is the likelihood that infection *establishes*? Quite often infection is already irreversible *under PrEP* (with PrEP incompletely blocking replication) and prior to the emergence of a latent reservoir. At the same time the virus may not yet be detectable in the blood plasma (e.g. at day 3 post-exposure; the eclipse period has been estimated to be 8-10 days in humans , PMID: 28365331). This irreversibility of infection under PrEP can be accurately modelled without the inclusion of a latent reservoir (Fig. 5 in the manuscript; compare also Fig. 4D in PMID: 29902179 and Fig. 3C in PMID: 3091848, which look at very small inocula).

On the other hand, I suspect the approach that authors proposed in the manuscript may have difficulties to handle complex mechanisms such as latent reservoir dynamics more realistically. Immune response is another complex piece in HIV, which is also critical but omitted in this model.
We agree that immune dynamics may be complex. However, the general numerical scheme can handle these (unlike most state-of-art stochastic simulation approaches):
(i) In our revised version we highlighted that the proposed numerical method can be applied to different models (i.e. previous comment; Suppl. Text S3).
(ii) The consideration of complex immune dynamics would alter the model, such that some reactions would become time-dependent functions. The proposed numerical method is suited to handle precisely that, as already demonstrated for time-dependent drug action on reaction rate a_5.
We are, however, very grateful for the authors comment and included a paragraph in the discussion (lines 488–492) regarding complex, possibly adaptive, immune dynamics shortly after exposure.

Reviewer #2: The paper by Zhang et al. explores different approximations to calculate the efficacy of prophylaxis strategies against HIV. The model for prophylaxis is based on viral dynamics and drug pharmacokinetics, and builds on the previous results from this group. Making those methods easily available through approximations is an important step to diffuse these approaches, and the paper is a nice contribution to the field. I have few comments for the

authors:

1. I think that the paper is a little long as it is. I would recommend (but worth discussing with the editor) to focus on one approximation in the main document, and to present comparisons with the two others in supp.

Thanks for pointing this out. We reorganized the article to focus only on the PGS method and hope that the article is now more appealing to the reviewer and a broader readership. Resulting major changes are highlighted at the beginning of this response letter.

2. It's great that the code is available. If you present your method as a way to empower clinicians/patients, I think it is important to make a shiny application available together with the manuscript? It's not a huge work but would increase its visibility

The authors share the reviewer's point of view (empowering patients and clinicians). An application is already envisaged, but may not be as trivial: Firstly, DTG is actually not recommended for PrEP currently. We use it as a test system w.r.t. the numerical method. For the suggested App, we will therefore focus on Truvada instead (which is approved and broadly used for PrEP). However, since Truvada is not the focus of this article, we decided not to include the App here.

3. Even if your study builds on previous works from your lab, it is important that the paper is self-standing. For instance it is difficult to judge the relevance of parameters from Table 1. At least you could show some descriptive statistics for your model predictions in Table 1 (in absence of any tx) : probability of infection according to exposure time (as shown in Table 3), viral kinetic profiles in case of successful infection ?

We rewrote parts of the method section, starting from the mathematical problem statement (chemical master equation) and then motivating the approach. In addition, we created a short section in the results ('*Model predictions in the absence of drugs*'), with a new Figure (Figure 2). Therein, we present model predictions (infection probabilities, time of viral extinction) and show how model predictions in the absence of drugs relate to clinically determined per-exposure infection risks (Fig. 2B).

4. It is unclear to me why you used plasma PK as a driver of efficacy in your model? There is no data in the literature on drug PK in compartments of interest? I would suspect that the drug PK in genital compartments in much less prone to high daily variations than in plasma

Thank you for this important remark, which we have addressed to some detail in a previous publication (PMID:29902179): As pointed out by the reviewer, our simulations do not take into account drug concentrations at the site of mucosal exposure (e.g. cervix, rectum, PMID: 22155899). These concentrations have, however, not been validated as targets for successful prevention or treatment, whereas data exist (albeit limited) for plasma drug concentrations. Instead, we modelled based on unbound concentrations, in line with the widely accepted 'free

drug hypothesis', stating that unbound concentrations at the target site are responsible for pharmacological action. For drugs highly bound to plasma protein (> 90%, PMID: 14585213), naturally since plasma protein concentrations are lower in tissues other than plasma, their *total* (bond + unbound) concentrations at sites other than the plasma are magnitudes lower (PMID: 22155899). Strikingly, however, the unbound plasma concentrations coincide with the unbound tissue concentrations (PMID:21633344), strongly arguing for the validity of the 'free drug hypothesis' (PMID: 21119731). Therefore, throughout the work, we assumed, according to the 'free drug hypothesis' that the unbound concentrations in plasma and at the target site coincide. Mathematically, the 'free drug hypothesis' was then implemented by 'correcting' the drugs' potency IC_{50} by protein binding (PMID:29902179). Note that dolutegravir is highly lipophilic (logP $\approx$ 2), enabling the unbound drug to rapidly cross cellular membranes, generating an equilibrium between the unbound drug on either side of the cellular membrane (PMID: 17899329).
Just for completeness: Nucleoside analogs (e.g. the compounds in Truvada) are highly lipophobic prodrugs that require active uptake and intracellular activation. For these compounds the 'free drug hypothesis' is not applicable.

5. Sometimes the extinction probability is calculated on V, T1, T2 (Fig 2). But sometimes the efficacy is calculated overall (e.g, Fig 3). It would be helpful if you could clearly define what is your criterion for extinction: one of the compartments, all the compartments? It would be important that you present first the 3 criterions, and then focus on one more specific.
The efficacy is always calculated overall, i.e. all viral compartments have to be cleared eventually (paragraph following eq. 2). The distinct equations (eq. 23) relate to the condition that the **exposure** originated from a single virus V, a single early infected cell T_1 or a single late infected cell T_2 (In essence: different initial conditions, but same dynamical system). From these equations (eq. 23), the extinction probabilities for any arbitrary initial conditions can be calculated (eq. 3) and from these extinction probabilities, prophylactic efficacy is derived (eq. 2 and eq. 1). Thanks for pointing this out and we clarified this in the revised manuscript (lines 220-5).
